# Detection of Nucleic Acids of the Fish Pathogen *Yersinia ruckeri* from Planktonic and Biofilm Samples with a CRISPR/Cas13a-Based Assay

**DOI:** 10.3390/microorganisms12020283

**Published:** 2024-01-29

**Authors:** Iván L. Calderón, M. José Barros, Nicolás Fernández-Navarro, Lillian G. Acuña

**Affiliations:** Laboratorio de RNAs Bacterianos, Departamento de Ciencias Biológicas, Facultad de Ciencias de la Vida, Universidad Andres Bello, Santiago 8370186, Chile; mariaj.barros@uandresbello.edu (M.J.B.); n.fernndeznavarro@uandresbello.edu (N.F.-N.)

**Keywords:** *Yersinia ruckeri*, fish pathogen, CRISPR/Cas13a-based assay, nucleic acids detection, biofilm and planktonic samples

## Abstract

*Yersinia ruckeri* is the cause of hemorrhagic septicemia, known as enteric redmouth disease, in salmonid fish species. This bacterial pathogen can form biofilms on abiotic surfaces of aquaculture settings or even on the surfaces of the fish themselves, contributing to their persistence in the aquatic environment. Detection methods for this and other fish pathogens can be time-consuming and lack specificity and sensitivity, limiting timely monitoring, the treatment of microbial infections, and effective control of their transmission in aquaculture settings. Rapid and sensitive detection methods for nucleic acids can be crucial for an appropriate surveillance of bacterial pathogens, and the CRISPR/Cas-based assays have emerged as a good alternative since it has been proven to be a useful tool for the rapid, specific, and sensitive detection of viruses and some bacteria. In this study, we explored the capability of the CRISPR/Cas13a system (SHERLOCK) to specifically detect both DNA and RNA (gene transcripts) from planktonic and biofilm samples of the bacterial fish pathogen *Y. ruckeri*. The assay was designed to detect the *gyrA* gene and the small noncoding RNAs (sRNAs) MicA and RprA from planktonic cultures and biofilm samples prepared in marine broth. The specific crRNA designed for these gene targets included a 28 nt specific gene sequence, and a scaffold sequence necessary for Cas13-binding. For all the assays, the nucleic acids obtained from samples were previously subjected to isothermal amplification with the recombinase polymerase amplification (RPA) method and the subsequent T7 transcription of the RPA amplicons. Finally, the detection of nucleic acids of *Y. ruckeri* was by means of a reporter signal released by the Cas13a collateral RNA cleavage triggered upon target recognition, measured by fluorescence- or lateral-flow-based readouts. This CRISPR/Cas13a-based assay was able to specifically detect both DNA and sRNAs from the *Y. ruckeri* samples, and the sensitivity was comparable to that obtained with qPCR analysis, highlighting the potential applicability of this CRISPR/Cas13a-based assay for fish pathogen surveillance.

## 1. Introduction

*Yersinia ruckeri* is an opportunistic pathogen responsible for enteric redmouth disease or yersiniosis, an infection that attacks both marine and freshwater fish. The disease is best characterized as hemorrhagic septicemia that mainly affects salmonids, and outbreaks have had an impact on salmon farms worldwide [1]. In aquaculture systems, *Y. ruckeri* has the ability to endure challenging conditions such antibiotics exposure related to the prevention and/or infection treatments of egg and fry phases of salmonid production [2]. However, this bacterium can survive and remain infective in these environments thanks to its capacity to form biofilms and grow on surfaces of fish tanks [3,4]. *Y. ruckeri* can persist for months in water sediments after an outbreak, in a dormant state, even retaining its virulence skills [5]. *Y. ruckeri* adheres with a high affinity to abiotic materials such as polystyrene, PVC, and wood, supports typically found in salmon farms (fish cages, tanks, and aquaculture buoys) [5,6].

In aquaculture settings, where bacterial, viral, fungal, and parasitic diseases threaten fish stocks, the early detection of pathogens becomes paramount for disease management and prevention. The timely identification of bacterial pathogens such as *Y. ruckeri* not only aids in minimizing disease outbreaks but also curtails environmental bacterial spread and reduces the indiscriminate use of antibiotics [7,8]. Current diagnostic methods for *Y. ruckeri*, including microbiological, serological, and molecular tests [9,10,11,12], generally demand fully equipped microbiological or molecular laboratory and skilled technicians, resulting in time lags of several hours or days from sample collection to pathogen detection [13,14]. Although field-applicable qPCR methods are available, their application requires equipment, time, and costs that limit the scale and scope of testing, monitoring, and processing by regulatory agencies.

To address these challenges, recent attention has turned to the CRISPR/Cas13a system (SHERLOCK), which has been demonstrated to have significant potential as a pathogen detection and diagnosis molecular tool [15,16,17,18]. The working principle of this CRISPR-Cas13a platform involves the detection of nucleic acids using the Cas13a enzyme which utilizes its trans-cleavage activity and the high specificity of its CRISPR RNA (crRNA) to the target detection. In this detection system, the first step combines reverse transcription with recombinase polymerase amplification in isothermal conditions, while the second step involves T7 transcription and CRISPR-Cas13a detection. Thus, this system comprises a Cas13 nuclease, the crRNA (interference RNA), and the single-stranded RNA target. This detection exploits the collateral unspecific RNase activity of Cas13 triggered upon target recognition and can use fluorescence- or lateral-flow-based readouts. Among the pathogens that have been tested for detection with CRISPR-Cas13a-based systems, we can mention viruses such as Zika virus, dengue virus, and SARS-CoV-2 [15,16,17]. Among bacteria, this system has proven to be useful for the detection of *Salmonella* spp. [19], *Chlamydia trachomatis* [20], *Neisseria gonorrhoeae* [21], *Yersinia pestis* [22], *Staphylococcus aureus* [23], among others. Parasites such as *Plasmodium falciparum* [24] and *Toxoplasma gondii* [25] have also been effectively identified with CRISPR/Cas13a-based systems, as well as tested for the diagnosis of fungal infections by *Aspergillus fumigatus* [26] and *Pneumocystis jirovecii* [27]. In addition to pathogens, the CRISPR/Cas13a system demonstrates versatility in the detection of various molecules or biomarkers such as microRNAs [28], and long non-coding RNAs (lncRNAs) [29] fall within its detectable range.

In the context of the detection and surveillance of salmonid bacterial pathogens, this study aimed to evaluate the ability to rapidly detect nucleic acids of the fish pathogen *Y. ruckeri*, from planktonic and biofilm samples, with high sensitivity and specificity, using this CRISPR/Cas13a-based assay and fluorescence- or lateral-flow-based readout methods. This detection assay was assessed for gDNA, total nucleic acids, and small noncoding RNAs (sRNAs), used as biomarkers, from both planktonic and biofilm bacterial samples of *Y. ruckeri* biotype 1, a strain which, being motile, favors the formation of biofilms. Significant results are presented below.

## 2. Materials and Methods Isolated from a Salmon Farm in Chile

### 2.1. Bacterial Strains and Culture Conditions

*Y. ruckeri* CD2, a strain isolated from a salmon farm in Chile [30], was routinely cultured at 26 °C in Trypticase soy broth (TSB) and aerated by shaking at 180 rpm in the case of planktonic cultures. *P. salmonis* was cultured in TSB supplemented (TSBs) with NaCl 3 g/L, fetal bovine serum (FBS) 2.5%, l-cysteine 0.05%, and FeCl_3_ 0.01 g/L and grown at 18 °C and 100 rpm for two days [31]. *S. Typhimurium* and *E. coli* were routinely grown at 37 °C in Luria–Bertani medium (LB) and aerated by shaking to 180 rpm. For biofilm cultures, *Y. ruckeri* was previously grown in TSB medium to stationary phase (OD_600_ 0.8) and then an inoculum of 2 mL was prepared with 1 × 10^8^ cells/mL in marine medium (BD) to incubate in a 24-well polystyrene microtiter plate without agitation at 26 °C for 24 h. In the case of *P. salmonis*, it was previously cultured for two days in TSBs medium and the and then an inoculum of 2 mL was prepared with 1 × 10^8^ cells/mL in marine medium (BD, Franklin Lakes, NJ, USA) to incubate in a 24-well polystyrene microtiter plate without agitation at 18 °C for two days. Cell counting was performed using a Neubauer chamber. After the incubation time of *Y. ruckeri* and *P. salmonis* biofilm cultures, the marine medium was removed, and the wells were washed with PBS and then with DEPC-treated water to then proceed with nucleic acid detection as described below. The bacterial strains used in this study are listed in Table S1 (Supplementary Material).

### 2.2. Antibiotic Treatment of Y. ruckeri Cultures

The antibiotic treatment of *Y. ruckeri* was carried out by supplementing planktonic cultures at OD_600_ 0.5 with oxytetracycline at a final concentration of 2 mg mL^−1^ and incubated for 30 min at 26 °C and aerated by shaking at 180 rpm.

After the incubation time of the *Y. ruckeri* and *P. salmonis* biofilm cultures, the marine medium was removed, and the wells were washed with PBS and then with DEPC-treated water. For DNA detection, the adherent bacteria were removed with a sterile swab, which was washed with 250 µL of DEPC-treated water. The samples were incubated for 10 min at 95 °C and centrifuged for 5 min at 10,000 rpm, and 4 µL of the supernatant was taken for detection using fluorescent Cas13 and lateral flow. For the detection of sRNAs, adherent bacteria washed with PBS were removed through low-frequency sonication, in the same PBS solution, with 3 pulses of 10 sec each, and the samples were centrifuged and total RNA was extracted using the GeneJET RNA Kit (Thermo Fisher, Waltham, MA, USA) following the manufacturer’s specifications.

### 2.3. Extraction of Total Nucleic Acids from Bacterial Lysates of Planktonic and Biofilm Samples

Total nucleic acids were obtained by incubating the planktonic and biofilm samples at 95 °C for 10 min in a dry bath. The sample was then centrifuged for 10 min at 10,000 rpm and 4 µL of the supernatant was used for nucleic acid detection.

### 2.4. Total RNA Extraction for Transcript Detection Assays

Total RNA extraction was performed using the GeneJET RNA Kit (Thermo Fisher, Waltham, MA, USA) following the manufacturer’s specifications. Briefly, the bacterial pellet obtained was resuspended in 100 µL of TE buffer supplemented with lysozyme (0.4 mg mL^−1^). It was incubated for 5 min at room temperature, and then 300 µL of lysis buffer was added, followed by stirring for 15 s. Next, 180 µL of ethanol (96–100%) was added and mixed by pipetting. The lysate was transferred to a column and centrifuged for 1 min at 12,000× *g*. The column was washed with 700 µL of Wash Buffer 1, followed by 600 µL and 250 µL of Wash Buffer 2. Finally, the RNA was eluted with 50 µL of DEPC-treated water. The gDNA was quantified using the QuantiFluor^®^ RNA System (Promega, Madison, WI, USA) and fluorescence was measured using a plate reader Biotek Synergy H1 (Agilent, Santa Clara, CA, USA) at 492 nm_Ex_/540 nm_Em_.

### 2.5. Reverse Transcription and Real-Time PCR (qRT-PCR)

Total RNA was treated with TURBO DNA-free™ kit to remove trace amounts of DNA. The cDNA was prepared from 10 and 1 ng total RNA using specific primers and Superscript II reverse transcriptase (Invitrogen, Carlsbad, CA, USA) according to the manufacturer’s instructions. The real-time PCR (qPCR) was performed as previously described by Calderón et al., 2014 [32] using KAPA SYBR^®^ FAST qPCR (KAPA Biosystems, Wilmington, MA, USA). Specific primers used are listed in Supplementary Table S1. The AriaMx Mx3000P equipment (Agilent, Santa Clara, CA, USA) was used. The Ct values obtained for each sample are reported. In parallel, to validate the results, the *gyrA* gene was amplified as a housekeeping gene. These experiments were repeated at least 3 times.

### 2.6. Bacterial Genomic DNA Extraction

For genomic DNA extraction, 5 mL of bacteria was grown in their respective media to stationary phase. Two milliliters of the culture were used for genomic DNA extraction and purification using the Wizard Genomic DNA Purification Kit (Promega, Madison, WI, USA) following the manufacturer’s specifications. The gDNA was quantified using the QuantiFluor^®^ ONE dsDNA System (Promega, Madison, WI, USA) and fluorescence was measured using a plate reader Biotek Synergy H1 (Agilent, Santa Clara, USA) at 504 nm_Ex_/531 nm_Em_.

### 2.7. crRNA and Primer RPA Design for DNA and RNA Detection

The crRNA design considers a 28 nt specific gene sequence (protospacer) that allows the specific detection of sRNAs MicA and RprA and the *gyrA* gene (gDNA) of *Y. ruckeri*.

The crRNA must be the reverse complement sequence of the target site in the transcribed RNA. In addition, this crRNA sequence contained a direct repeat sequence added at the 5′ end (DR, 5′-GGGGAUUUAGACUACCCCAAAAACGAAGGGGGGACUAAAAC-3′) [33], required for crRNA recognition by Cas13. Furthermore, to synthesize crRNA through in vitro transcription, the T7 polymerase promoter sequence (5′-TAATACGACTCTCACTATA-3′) was finally added to the 5′ end of the DR-crRNA sequence. The designed crRNA was purchased as ssDNA oligonucleotide from Integrated DNA Technologies (IDT, Coralville, IA, USA). The spacer sequence used for each gene is shown in Table S2 (Supplementary Material). The ssDNA was used as template to synthesize the crRNA through in vitro transcription using the HiScribe T7 kit (NEB, Ipswich, MA, USA) following the manufacturer’s specifications.

On the other hand, primers compatible with recombinase polymerase amplification (RPA) were designed considering the standardized parameters previously described [33], such as primer length between 25 and 35 bp, GC% content between 20 and 80%, and a Tm between 54 and 67 °C. Additionally, the T7 polymerase promoter sequence (5′-AATTCTAATACGACTCACTATAGGGTCCA-3′) was added to the 5′ end of each forward primer.

### 2.8. Detection of Y. ruckeri Nucleic Acids with the CRISPR/Cas13a-Based Assay

Detection of nucleic acids was performed in a one-pot reaction, according to a previously described SHERLOCK protocol [15,33], which includes a pre-amplification step with recombinase polymerase amplification (RPA), which can isothermally amplify either RNA (RT-RPA) or DNA, and introduce a T7 RNA polymerase promoter, allowing RNA transcription and subsequent detection by the *Leptotrichia wadei* ortholog of Cas13a (*lw*Cas13a). Detection of target RNA was performed by the Cas13a collateral RNA cleavage-mediated release of reporter signal. The pC013—Twinstrep-SUMO-huLwCas13a was a gift from Feng Zhang (Addgene plasmid # 90097; http://n2t.net/addgene:90097; accessed on 20 December 2023; RRID: Addgene_90097).

RPA reagents from the TwistAmp Basic Kit (TwistDx, Maidenhead, UK) and TwistAmp^®^ Basic RT (TwistDx, Maidenhead, UK) were used. A Biotek Synergy H1 microplate reader (Agilent, Santa Clara, USA) was preheated to 37 °C. The template was added at a volume of 4 µL into 20.6 µL of the detection mix.

For the fluorescence-readout method, the final reaction mixture was homogenized and centrifuged at 500× *g* for 30 s before 20 µL was pipetted into a 96-well black optical plate (Nunc™ MicroWell™, Thermo Fisher, Waltham, MA, USA). The plate was loaded into the microplate reader and fluorescence readings at 30 min were carried out at 485 nm_Ex_/528 nm_Em_. For fluorescence detection of nucleic acids, the fluorescence background corresponding to the negative control (containing all reagents except bacterial sample) was subtracted and final values are presented as arbitrary units (AU) of fluorescence. The experiments were repeated three times.

For lateral flow (immunochromatographic)-based readouts, a lateral flow reporter flanked by fluorescein and biotin on separate ends was utilized. This reporter was synthesized at IDT (5′/56-FAM/mArArU rGrGrC mAmArA rUrGrG rCmA/3Bio/-3′). On the lateral flow strips, there is a streptavidin line where it will bind to biotin, and anti-fluorescein antibodies labeled with gold nanoparticles will only bind when RNA reporters are cleaved due to the collateral activity of Cas13. The lateral flow reading was performed using the universal lateral flow dipstick kit for the detection of biotin- and FITC-labeled analytes (HybriDetect, Milenia Biotec, Gießen, Germany). An amount of 20 µL of detection mix was mixed with 80 µL of analyte-specific solution, and the reaction was incubated for 5 to 10 min by placing the dipsticks in the tube where the reaction was performed at room temperature.

### 2.9. Statistics

The data were statistically analyzed with Student’s *t*-test. Values of *p* < 0.05 were considered statistically significant.

## 3. Results

### 3.1. Detection of Y. ruckeri DNA from Biofilm Samples Using a CRISPR/Cas13a-Based Assay

**Figure 1:**
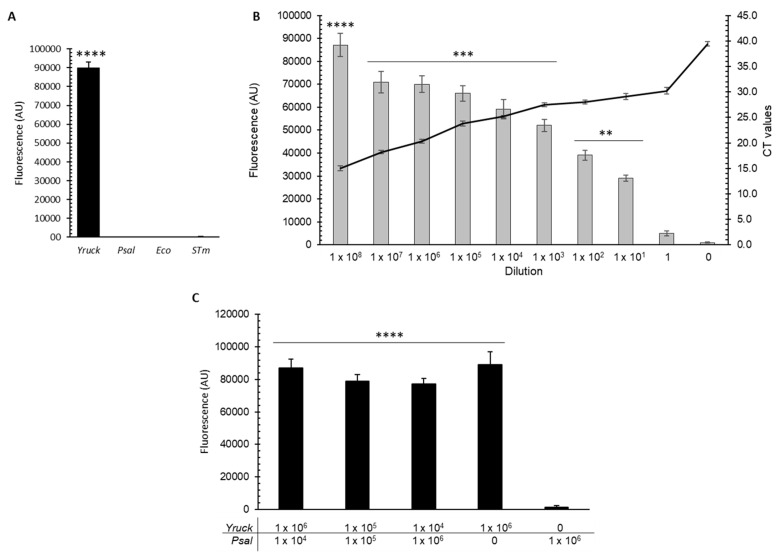
Detection of *Y. ruckeri* DNA from planktonic samples using a CRISPR/Cas13a-based assay. (**A**) CRISPR/Cas13a-based assay from 1 ng of purified gDNA of *Y. ruckeri* (*Yruck*), *P. salmonis* (*Psal*), *E. coli* (*Eco*), and *S*. Typhimurium (*STm*), respectively. RPA was performed from each gDNA, and the detection was performed through fluorescence-based readouts. (**B**) CRISPR/Cas13a-based assay from total nucleic acids of bacterial lysates serially diluted from an initial planktonic culture of 1 × 10^8^ cell/mL of *Y. ruckeri*. The bars show the measured fluorescence, and the line shows the Ct value obtained from a qPCR assay performed with the same samples. (**C**) CRISPR/Cas13a-based assay from mixtures of bacterial lysates from planktonic cultures of *Y. ruckeri* and *P. salmonis*. Dilutions were prepared at 1 × 10^6^, 1 × 10^5^, and 1 × 10^4^ cell/mL and 500 µL of each dilution was mixed as indicated in the figure. The mixtures were then incubated at 95 °C for bacterial lysis and subsequent detection of *Y. ruckeri* by fluorescence. From each of these lysates, 4 µL was used for detection analysis. AU: arbitrary units. Asterisks represent statistical differences with respect to the control or blank (** *p* < 0.01, *** *p* < 0.001, and **** *p* < 0.0001). Data represent the means ± standard deviations.

The gene target for the 28 nt sequence included in the crRNA design for specific detection of *Y. ruckeri* DNA was *gyrA* (crRNA*gyrA*, see Section 2), a conserved gene among bacteria, including those used in this study as controls. Firstly, to evaluate the functionality of the crRNA designed to detect DNA of *Y. ruckeri*, 1 ng of purified gDNA from *Y. ruckeri* was used as the sample. As negative controls, gDNA purified from the fish pathogen *Piscirickettsia salmonis* was used, as well as from bacteria belonging to the same family of *Y. ruckeri* (*Enterobactericeae*), namely *Escherichia coli* and *Salmonella* Typhimurium, whose respective *gyrA* gene sequences have greater similarity. As shown in Figure 1A, through fluorescence-based readouts, only the sample from *Y. ruckeri* was detected, demonstrating the functionality and specificity of the CRISPR/Cas13a-based assay and the designed crRNA (crRNA-*gyrA*).

We proceeded to evaluate whether this platform was effective in detecting *Y. ruckeri* from bacterial lysates, including serial dilutions of these samples. Fluorescence readouts were obtained in all the dilutions of the samples containing *Y. ruckeri*, over the background of the negative control not containing bacterial samples, demonstrating that this detection assay is also effective and specific with bacterial lysates (Figure 1B). In addition, the fluorescent detection signal was inversely proportional to the serial dilutions and, at the same time, this assay proves to be highly sensitive, equivalent to that of a qPCR assay, as evidenced by the Ct values obtained at the highest dilution compared to the negative control (Figure 1B). Then, we evaluated the capability of the platform to detect *Y. ruckeri* from mixed bacterial lysates of planktonic cultures, using the marine fish pathogen *P. salmonis*. Fluorescence readouts were also obtained only from the samples containing *Y. ruckeri*, demonstrating that this detection assay is also effective for *Y. ruckeri* identification in samples containing the target and non-specific bacteria at different dilutions (Figure 1C).

### 3.2. Detection of Y. ruckeri DNA from Biofilm Samples Using a CRISPR/Cas13a-Based Assay

**Figure 2:**
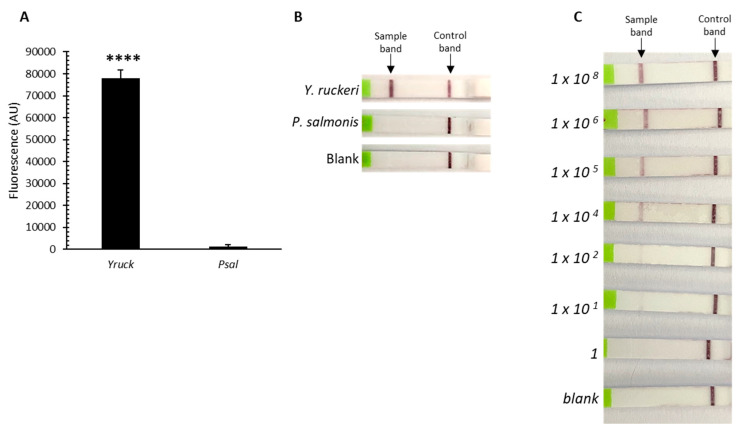
Detection of *Y. ruckeri* DNA from biofilm samples using a CRISPR/Cas13a-based assay. (**A**) CRISPR/Cas13a-based assay from biofilm samples of *Y. ruckeri* and *P. salmonis* obtained from an inoculum of 1 × 10^8^ cells/mL incubated with marine medium (BD) in a 24-well polystyrene microtiter plate without agitation at 26 °C for 24 h, and at 18 °C for 48 h for *Y. ruckeri* and *P. salmonis*, respectively. The detection was performed through fluorescence-based readouts from lysed bacteria obtained from biofilms. AU: arbitrary units. (**B**) CRISPR/Cas13a-based assay from biofilm samples of *Y. ruckeri* and *P. salmonis* obtained as in A. The detection was performed through lateral-flow-based readouts from lysed bacteria obtained from biofilms. Sample band corresponds to the biofilm sample. (**C**) CRISPR/Cas13a-based assay from biofilm samples of *Y. ruckeri* obtained from different initial bacterial inocula (1 × 10^n^ cell mL^−1^). The detection was performed through lateral-flow-based readouts from lysed bacteria obtained from biofilm samples. Asterisks represent statistical differences with respect to the control (**** *p* < 0.0001). Data represent the means ± standard deviations.

We also evaluated the capability of the platform to detect *Y. ruckeri* from biofilms formed in marine broth, using *P. salmonis* as the control. Fluorescence readouts were also only obtained from the samples of *Y. ruckeri* biofilms (Figure 2A), demonstrating that this detection assay is also effective in conditions mimicking the aquaculture settings, because this bacterium can form biofilms in salmon farms, which presents an additional challenge for the detection and control of this fish pathogen. We then tested a lateral-flow readout method, allowing for visual detection on commercial lateral flow strips, as previously reported [16]. The specific detection was possible from biofilm samples in <70 min (Figure 2B), evidencing a high sensitivity as demonstrated by the significant fluorescence signal even from the biofilm sample obtained from the lowest initial bacterial inoculum (1 × 10 cells/mL) (Figure 2C).

### 3.3. Detection of Y. ruckeri Transcripts (sRNAs) from Bacterial Lysates of Planktonic and Biofilm Cultures Using a CRISPR/Cas13a-Based Assay

**Figure 3:**
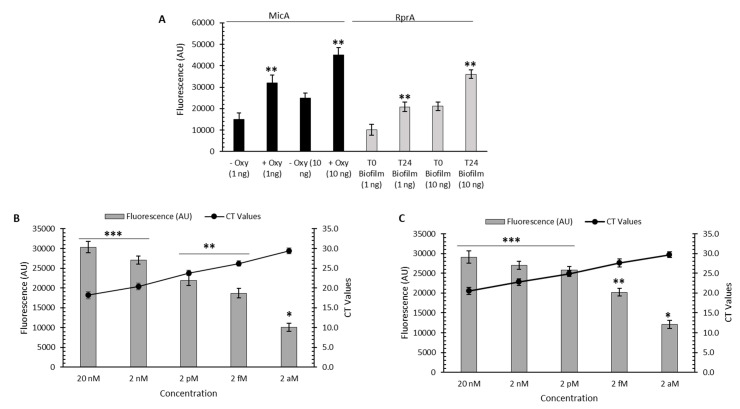
Detection of *Y. ruckeri* transcripts (sRNAs) from bacterial lysates of planktonic and biofilm cultures using a CRISPR/Cas13a-based assay. (**A**) CRISPR/Cas13a-based assay from total RNA (1 ng or 10 ng) extracted from *Y. ruckeri* planktonic cultures treated (+) or not (−) with oxytetracycline (Oxy) to detect the expression of the MicA sRNA, and *Y. ruckeri* biofilm cultures of 24 h (T24) to detect the expression of RprA sRNA. (**B**) Transcript detection of the MicA sRNA through the CRISPR/Cas13a-based assay (bars) and RT-qPCR (line) from different RNA concentration templates obtained from the total RNA extracted from *Y. ruckeri* planktonic cultures treated with Oxy. (**C**) Transcript detection of the RprA sRNA through the CRISPR/Cas13a-based assay (bars) and RT-qPCR (line) from different RNA concentration templates obtained from the total RNA extracted from *Y. ruckeri* biofilm cultures of 24 h. Asterisks represent statistical differences with respect to the control or blank (* *p* < 0.05, ** *p* < 0.01, *** *p* < 0.001). Data represent the means ± standard deviations.

To also evaluate the CRISPR/Cas13a-based assay for the detection of *Y. ruckeri* transcripts from planktonic and biofilm samples, we designed crRNA to specifically detect sRNAs used as markers of stress (antibiotic exposure) and physiological responses (biofilm formation) in *Y. ruckeri*. Thus, the platform was used to detect the expression of the MicA sRNA (CRISPR/Cas13a/crRNA-MicA platform), from *Y. ruckeri* planktonic cultures treated with oxytetracycline, and of the RprA sRNA (CRISPR/Cas13a/crRNA-RprA platform), from *Y. ruckeri* biofilm samples. From the total RNAs extracted from these samples, an isothermal amplification process of a specific sequence of the sRNAs to be detected was performed, using the RT-RPA technique. Then, we proceeded with the in vitro transcription of these RT-RPA amplicons. Finally, the MicA and RprA sRNAs were specifically detected from planktonic and biofilm samples, respectively, using the fluorescent readout method (Figure 3A). As sensitivity controls, the expression induction of MicA and RprA was also analyzed using qRT-PCR (Figure 3B,C) and represented as Ct values. To determine the detection limit of this assay, various dilutions of the extracted total RNAs were used, which were then treated in the same way as the undiluted total RNAs. As shown in Figure 3B,C, comparing fluorescence-based readouts and Ct values, the sensitivity of the CRISPR/Cas13a/crRNA-sRNAs platforms were once again comparable to that of a qRT-PCR analysis, being able to detect a total RNA concentration of 2 aM used as the initial template.

## 4. Discussion

The fish bacterial pathogens pose significant challenges to the aquaculture industry due to their impact on fish populations and the difficulty in detection from aquaculture settings and in the control of its spread. *Y. ruckeri* can lead to acute infection and disease during hatchery and sea-transfer stages [34], causing high mortality rates in salmonids, resulting in significant economic losses in salmonid farming [35]. Salmonids infected with *Y. ruckeri* can carry the bacteria as a latent infection for several months, potentially facilitating cryptic spread between facilities that exchange fish [36]. In this context, rapid and sensitive detection methods for this and other fish pathogens can be crucial for appropriate surveillance in aquaculture. The detection methods currently used have several limitations, for example, the lack of sensitivity and specificity, and the time-consuming or the slow speed of the culture-dependent methods [37]. In this sense, rapid and sensitive detection methods for nucleic acids, even without major requirements for specialized equipment, could be crucial for an appropriate surveillance of bacterial pathogens. This study aimed to evaluate the ability of a CRISPR/Cas13a-based assay to rapidly detect nucleic acids of the fish pathogen *Y. ruckeri*, with high specificity and sensitivity (Figure 1, Figure 2 and Figure 3), and even with the potential to be implemented in fish farms without the need for specialized and expensive analytical equipment (Figure 2). To our knowledge, this CRISPR-Cas13a system had not been used to detect nucleic acids of this bacterial fish pathogen, let alone from biofilm samples, a feature associated with salmon farming concerns. In addition, we also tested this system to detect sRNAs (Figure 3), exploring the possibility of monitoring them as biomarkers of specific processes or abilities of this pathogen, such as biofilm formation and antibiotic resistance [38].

Based on the Cas13a-based molecular detection platform developed by the Zhang group [15], we demonstrated that our CRISPR/Cas13a/crRNA-*gyrA* platform can be used as a specific assay to detect *Y. ruckeri* from gDNA (Figure 1), and from bacterial lysates of planktonic samples (Figure 1B), including mixed bacterial lysates of co-occurring bacteria in salmonid farms (Figure 1C). The sensitivity of this assay was comparable to qPCR analysis, detecting *Y. ruckeri* even in the most diluted planktonic sample by both methods. A CRISPR/Cas12a-based assay performed for detection of the fish pathogen *Renibacterium salmoninarum* also exhibited higher sensitivity [39]; however, both studies are not entirely comparable, because they were performed with different DNA samples and reporter systems, based on the different activities of Cas12a and Cas13a enzymes. One advantage we could mention about the use of Cas13 over Cas12 in the detection of nucleic acids is that the Cas13 enzyme employed in this detection platform does not require stringent preferences in terms of sequence at the target site, whereas the Cas12 enzyme necessitates the presence of a protospacer-adjacent motif to proceed with excision. This allows for greater flexibility and a wider range of targets in platforms using Cas13 compared to those using Cas12 [33]. Another advantage of Cas13 over Cas12 in nucleic acid platforms is its relatively robust collateral activity, making it a more robust tool for the detection of nucleic acids [40].

Using isothermal amplification with RPA (recombinase polymerase amplification), this detection platform works at a relatively low and constant temperature (generally ranging from 37 to 42 °C), thereby significantly surpassing the swiftness of conventional PCR methods. RPA has been reported for rapid and sensitive DNA detection with other pathogens, but alone, it is not sensitive enough to detect low levels of DNA [15,16]. In addition, RPA shows reduced sensitivity to inhibitors present in intricate sample matrices, in comparison to other amplification methodologies, and can be tailored for diverse applications extending beyond DNA amplification, including RNA detection and other nucleic acid-based assays, thereby expanding its utility in the realms of diagnostics and research. Likewise, the RPA-CRISPR/Cas13a system enhances its accessibility and adaptability for implementation in various scenarios, including point-of-care diagnostics, rendering it suitable for utilization in field applications, remote regions, or settings with limited resources, where access to laboratory infrastructure is restricted. This could potentially aid in the containment of disease transmission among the hatchery or aquaculture fish stock populations and minimize the transmission of this pathogen to wild fish populations. Additionally, it has the potential to optimize the utilization of antibiotics by identifying periods when bacteria are absent, thus reducing the unnecessary administration of antibiotics that may contribute to the proliferation of multiple-drug-resistant bacteria [8,39].

The formation of biofilms adds an extra layer of complexity to managing this pathogen. *Y. ruckeri* can form biofilms in aquaculture settings. Biofilms are microbial communities attached to biotic and abiotic surfaces and it is well recognized that most aquatic bacteria are associated with surfaces rather than in the planktonic state, and *Y. ruckeri* is not the exception [6,41]. *Y. ruckeri* can persist for months in water sediments after an outbreak, in a dormant state, even retaining its virulence skills [5]. *Y. ruckeri* adheres with a high affinity to abiotic materials such as polystyrene, PVC, and wood, supports typically found in salmon farms (fish cages, tanks, and aquaculture buoys), forming stable biofilms [6,42]. The formation of biofilms by *Y. ruckeri* may contribute to its survival because it provides a protective environment for the bacteria, allowing them to resist adverse conditions such as the exposure to disinfectants used in the aquaculture industry. The presence of biofilms can also facilitate the transmission of *Y. ruckeri* among fish and contribute to the persistence of infection in aquaculture facilities, acting as reservoirs of bacteria, facilitating the re-infection of fish populations even after cleaning and disinfection measures. In this sense, it is crucial to develop a molecular tool that allows the simple, rapid, specific, and sensitive detection of this pathogen from biofilms. Thus, using our CRISPR/Cas13a/crRNA*gyrA* platform, we were able to detect *Y. ruckeri* also from biofilm samples, formed under conditions that mimic a seawater environment, with a simple method of extraction from adherent bacteria from polystyrene with a sterile swab and the subsequent extraction of total nucleic acids through temperature lysis in a few minutes. Keeping in mind the need to develop a potentially applicable assay without further requirements of specialized equipment, in this case, in addition to using the fluorescence-based detection system, we tested a colorimetric detection method that would allow a rapid visual inspection. Thus, by coupling the CRISPR/Cas13a/crRNA*gyrA* platform to a lateral flow assay kit (HybriDetect, Milenia Biotec), we were able to detect, also specifically and with high sensitivity, *Y. ruckeri* from biofilm samples (Figure 2). This lateral-flow-based method, coupled to the CRISPR/Cas system, had already been tested with high sensitivity in the detection and identification of other types of samples and organisms [16,43], but had not been tested on biofilm samples of fish pathogens. Developing effective detection and diagnostic tools based on this platform could be crucial to minimize the impact of *Y. ruckeri* on fish populations and improve overall aquaculture management to reduce the transmission of this and other fish pathogens. Although *Y. ruckeri* is not the most prevalent pathogen in aquaculture globally, the immunosuppression of salmonids caused by other bacteria, viruses, fungi, and/or algal blooms could render the host more susceptible to secondary infections caused by this opportunistic pathogen. For example, it is well known that pathogens such as *Aeromonas salmonicida* and salmonid alphavirus have been found to disrupt the microbiota of salmonids, leading to dysbiosis and an increase in opportunistic pathogens [44,45]. This alteration of the microbiota can render the host more susceptible to secondary bacterial infections [46]. Additionally, gill disorders caused by fish poxviruses have been shown to negatively impact respiratory and osmoregulatory functions, leading to immunosuppression and increased susceptibility to secondary infections [47]. On the other hand, certain harmful algal blooms can affect gill functions in fish and increase their susceptibility to secondary bacterial infections [47].

Since CRISPR/Cas13a specifically recognizes single-stranded RNAs, the RPA-CRISPR/Cas13a system can be tailored for diverse applications extending beyond DNA amplification, including RNA detection and other nucleic acid-based assays, thereby expanding its utility in the realms of diagnostics and research [18]. Thus, in this study, we also demonstrated that the CRISPR/Cas13a platform is also useful to analyze or monitor the expression of specific *Y. ruckeri* transcripts, for example, those that can be used as biomarkers of certain physiological responses or stress conditions. Thus, we compared the sensitivity of this platform with RT-qPCR analysis to detect the expression of RprA sRNA, induced upon exposure to oxytetracycline, and the expression of MicA sRNA, induced in the biofilm formation process of this pathogen. Although the detection sensitivity is comparable between the two methods, the processing time and the time required to obtain the result were significantly reduced with the CRISPR/Cas13a platform, which can be critical when analyzing certain virulence markers to make timely decisions regarding surveillance and treatments associated with this or other pathogens. The limitations of the present study lie mainly in the fact that these assays, although they were performed with samples that mimic certain conditions associated with the growth of this bacterium and its environment in fish farms, did not consider all the factors of an environmental sample, which can result in the involvement of interfering elements that may affect the nucleic acids’ stability, amplification process, and/or detection. Despite these limitations, the RPA/CRISPR/Cas platform shows promise in the specific, sensitive, and rapid detection of fish pathogens, and further research and development may help analyze and overcome these challenges.

## Data Availability

The data presented in this study are available on request from the corresponding author.

## Supplementary Materials

The following supporting information can be downloaded at https://www.mdpi.com/article/10.3390/microorganisms12020283/s1. Table S1: Bacterial strains used in this study [30,48,49], Table S2: Primers (Oligos) used in this study, Table S3: crRNAs used in this study.
